# Novel Protective Role of Myeloid Differentiation 1 in Pathological Cardiac Remodelling

**DOI:** 10.1038/srep41857

**Published:** 2017-02-06

**Authors:** Xiaojv Xiong, Yu Liu, Yang Mei, Jianye Peng, Zhiqiang Wang, Bin Kong, Peng Zhong, Liang Xiong, Dajun Quan, Qi Li, Guangji Wang, He Huang

**Affiliations:** 1 Department of Cardiology, Renmin Hospital of Wuhan University, Wuhan, Hubei Province, PR China; 2 Cardiovascular Research Institute of Wuhan University, Wuhan, Hubei Province, PR China; 3 Hubei Key Laboratory of Cardiology, Wuhan 430060, Hubei Province, PR China

## Abstract

Myeloid differentiation 1 (MD-1), a secreted protein interacting with radioprotective 105 (RP105), plays an important role in Toll-like receptor 4 (TLR4) signalling pathway. Previous studies showed that MD-1 may be restricted in the immune system. In this study, we demonstrated for the first time that MD-1 was highly expressed in both human and animal hearts. We also discovered that cardiac-specific overexpression of MD-1 significantly attenuated pressure overload-induced cardiac hypertrophy, fibrosis, and dysfunction, whereas loss of MD-1 had the opposite effects. Similar results were observed for *in vitro* angiotensin II-induced neonatal rat cardiomyocyte hypertrophy. The antihypertrophic effects of MD-1 under hypertrophic stimuli were associated with the blockage of MEK-ERK 1/2 and NF-κB signalling. Blocking MEK-ERK 1/2 signalling with a pharmacological inhibitor (U0126) greatly attenuated the detrimental effects observed in MD-1 knockout cardiomyocytes exposed to angiotensin II stimuli. Similar results were observed by blocking NF-κB signalling with a pharmacological inhibitor (BAY11–7082). Our data indicate that MD-1 inhibits cardiac hypertrophy and suppresses cardiac dysfunction during the remodelling process, which is dependent on its modulation of the MEK-ERK 1/2 and NF-κB signalling pathways. Thus, MD-1 might be a novel target for the treatment of pathological cardiac hypertrophy.

Cardiac hypertrophy is a complex remodelling process of the heart that is induced by physiological or pathological stimuli, including hypertension, valve disease, myocardial ischaemia, and genetic mutations. It is characterized by an increase in the size of individual cardiac myocytes and whole-organ enlargement^1^,^2^. Whereas cardiac hypertrophy can function as an adaptive mechanism by which the heart responds to stressful conditions, prolonged and severe hypertrophy can lead to poor clinical outcomes, including arrhythmia, sudden death, and heart failure^3^,^4^. Although the pathways that promote hypertrophic responses have been extensively investigated, the mechanisms underlying these pathways and their antagonism have not been as clearly defined. Thus, it is important to clarify the molecular mechanisms involved in maladaptive cardiac remodelling to identify effective therapeutic targets for suppressing cardiac hypertrophy.

Toll-like receptors (TLRs) are the primary receptors of the innate immune system, and the associated signalling pathway has been shown to play an essential role in the induction of immune responses^5^,^6^. TLR4, one of the most important TLR family receptors, participates in two classical signalling pathways: the myeloid differentiation factor 88 (MyD88)-dependent and MyD88-independent pathways. These signalling pathways activate the mitogen-activated protein kinase (MAPK) and nuclear factor kappa B (NF-κB) pathways, which have been suggested to contribute to the development of pathological cardiac hypertrophy^7^,^8^. Recent evidence suggests that TLR4-mediated signalling is involved in several cardiovascular diseases, including atherosclerosis, ischaemia/reperfusion injury, and cardiac remodelling^9^,^10^,^11^. For example, TLR4-mutant mice have been shown to be resistant to ischaemia-triggered cardiac injury^12^, and the cardiac-specific expression of TLR4 has been demonstrated to be upregulated in cardiomyopathy^13^. Moreover, in preclinical studies, TLR4 has been shown to play important roles in both left ventricular (LV) remodelling and the amelioration of functional impairments following myocardial infarction (MI)^14^.

Myeloid differentiation 1 (MD-1, also known as lymphocyte antigen 86, Ly86) is a secreted glycoprotein that forms a complex with radioprotective 105 (RP105)^15^,^16^. RP105 is a TLR homologue that has been shown to regulate TLR4 signalling. It is structurally similar to TLR4 but lacks an intracellular Toll/interleukin receptor (TIR) signalling domain. RP105-MD-1 has been demonstrated to be an important TLR4 regulator. Two possible mechanisms for the inhibition of TLR4-MD2 (Myeloid Differentiation-2) by RP105-MD-1 were proposed previously: lateral binding of TLR4-MD2 to the RP105-MD1 complex, or formation of the TLR4-MD2/RP105-MD1 complex, resembling the usual ligand-induced TLR homodimers^17^. Therefore, activation of the TLR4-MD2 heterodimer by a ligand results in activation of the intracellular signalling domain, thereby initiating a downstream signalling cascade. Formation of the unusual 2:2 homodimer by TLR4-MD2 and RP105-MD1 results in alteration of the TLR4 signalling cascade^18^. Recent studies have reported that the activation of TLR4 is involved in several cardiovascular diseases, and RP105 is a known inhibitor of the TLR4 signalling pathway^16^,^19^ that also plays an essential role in cardiovascular diseases. Recent evidence suggests that RP105 protects against myocardial ischaemia-reperfusion injury by suppressing the TLR4 signalling pathways in rat models^20^,^21^ and that RP105 deficiency aggravates cardiac dysfunction after MI in mice^22^. MD-1 is an indispensable accessory molecule that is required for the cell surface expression of RP105^15^,^23^,^24^, but its role as a regulator of cardiac hypertrophy and fibrosis has not previously been explored. In the present study, we have shown that induction of the constitutive cardiac expression of human MD-1 in mice confers protection against cardiac hypertrophy and fibrosis by blocking MEK-ERK1/2 and NF-κB signalling and that MD-1^−/−^ mice display the opposite phenotype in response to pressure overload. The results of our experiments using cardiac-specific transgenic (TG) MD-1 and MD-1^−/−^ mice have suggested that MD-1 is a crucial modulator of cardiac remodelling and heart failure.

## Results

### MD-1 expression is down-regulated in human HCM hearts and murine hypertrophic hearts

**Figure 1:**
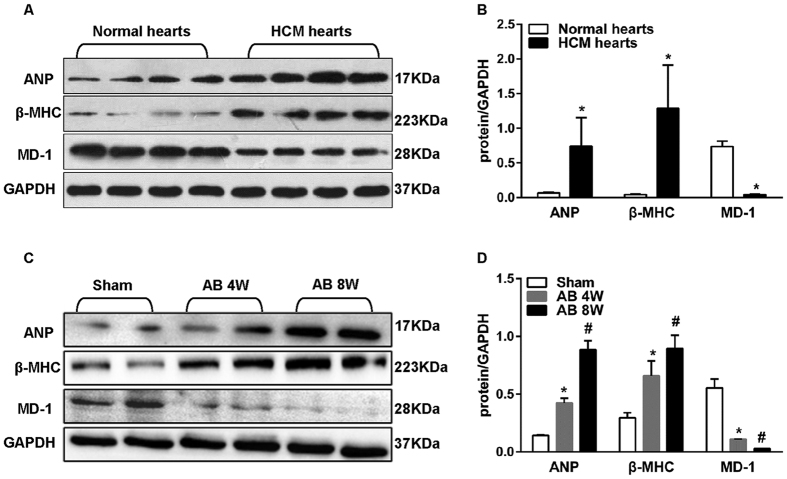
ANP, β-MHC, and MD-1 expression in human failing hearts and experimental hypertrophic models. (**A**,**B**) ANP, β-MHC, and MD-1 protein levels in samples obtained from donor hearts and HCM hearts (n = 6 samples per group). (**C**,**D**) Representative western blots of ANP, β-MHC, and MD-1 in mouse hearts at the indicated time points after AB (n = 8). (**A**,**C**) Representative blots. (**B**,**D**) Quantitative results. *P < 0.05 vs. normal donors or shams; ^#^P < 0.05 vs. sham.

To investigate the potential role of MD-1 in cardiac hypertrophy, we first examined MD-1 expression in the left ventricles of hypertrophic cardiomyopathy (HCM) patients who had undergone heart transplantation due to end-stage heart failure. Western blot analyses showed that the MD-1 protein levels were decreased by 94 ± 1% in failing hearts (n = 6) compared with donor hearts (n = 6) and that these decreases were accompanied by increases in the levels of proteins encoded by foetal genes, including atrial natriuretic peptide (ANP) and β-myosin heavy chain (β-MHC) (Fig. 1A,B). Similarly, the MD-1 protein levels progressively decreased in the murine hearts after 4–8 weeks of aortic banding (AB) treatment, but the levels of proteins encoded by foetal genes, including ANP andβ-MHC, increased during the same period (Fig. 1C,D). These data potentially implicate MD-1 in cardiac hypertrophy and cardiomyopathy.

### MD-1 negatively regulates Ang II-induced myocyte hypertrophy

**Figure 2:**
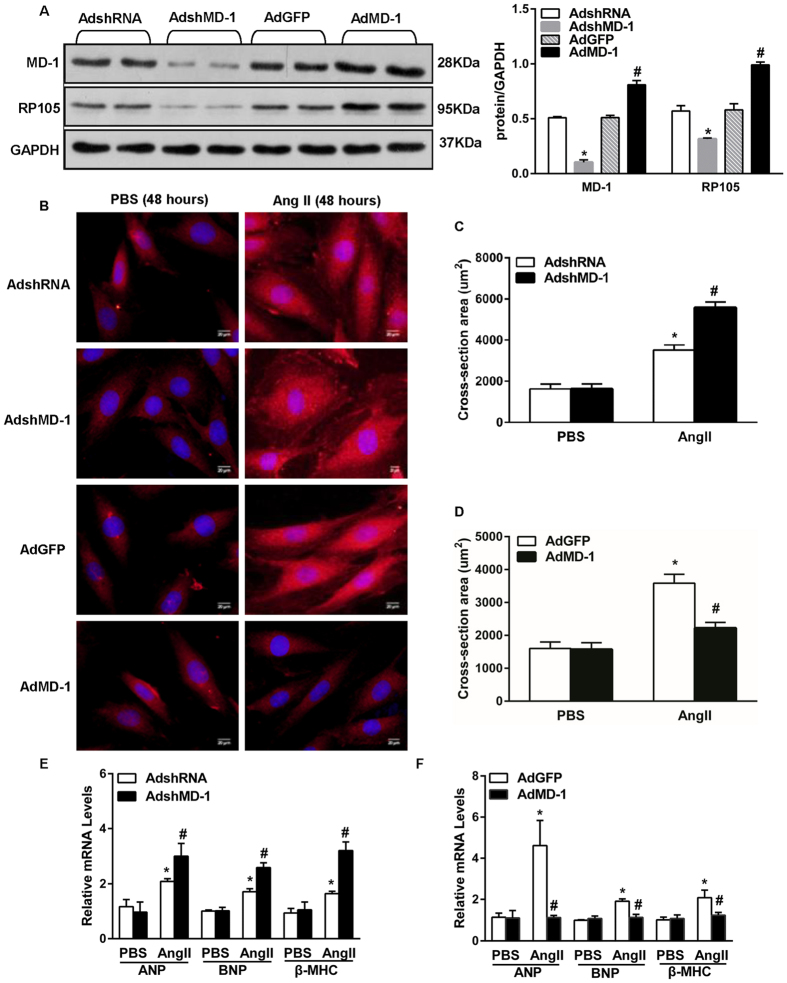
The effects of MD-1 and RP105 on hypertrophy *in vitro*. (**A**) The MD-1 and RP105 protein levels after infection with AdshMD-1 or AdMD-1 (n = 4). (**B**) Representative image of cultured NRCMs infected with AdshRNA, AdshMD-1, AdGFP or AdMD-1 and treated with Ang II. (**C**,**D**) Results of quantitative analysis of the cell surface areas for the indicated groups. n = 4, *P < 0.05 vs. PBS controls; ^#^P < 0.05 vs. AdshRNA/Ang II or AdGFP/Ang II. More than 100 cells were analysed for each group. (**E**,**F**) RT-PCR analysis of hypertrophy markers (ANP, BNP and β-MHC) in neonatal rat cardiomyocytes infected with AdshMD-1 (**E**) or AdMD-1 (**F**) following treatment with PBS or Ang II for 48 h. Similar results were obtained from three independent experiments. *P < 0.05 vs. PBS controls; ^#^P < 0.05 vs. AdshRNA/Ang II or AdGFP/Ang II.

To examine the *in vitro* role of MD-1 in hypertrophy, we performed gain- and loss-of-function studies using cultured neonatal rat cardiomyocytes (NRCMs). Cells were infected with AdMD-1 or AdshMD-1. Infection with AdMD-1 resulted in a substantial increase in the MD-1 protein level in NRCMs, whereas infection with AdshMD-1 led to a marked decrease in this level (Fig. 2A). In addition, we measured the protein level of RP105, the expression of which depends on MD-1. In accordance with the MD-1 expression results, infection with AdMD-1 resulted in an increase in the RP105 protein level in NRCMs, whereas infection with AdshMD-1 led to a marked decrease in this level (Fig. 2A). Further experiments revealed that either reducing or elevating the MD-1 level did not result in a difference in cardiomyocyte size between cells grown under experimental and basal conditions (phosphate-buffered saline (PBS)). However, exposure to angiotensin II (Ang II;  1 uM) for 48 h resulted in Ang II-induced hypertrophy; this effect was aggravated in MD-1-knockdown myocytes (Fig. 2B,C) and alleviated in MD-1-overexpressing cells (Fig. 2B,D), as indicated by analyses of cell surface area (CSA) (Fig. 2B–D) and mRNA levels of the hypertrophy markers ANP, B-type natriuretic peptide (BNP) and β-MHC (Fig. 2E,F). These results indicate that MD-1 negatively regulates Ang II-induced myocyte hypertrophy.

### Loss of MD-1 exaggerates pressure overload-induced cardiac hypertrophy *in vivo*

**Figure 3:**
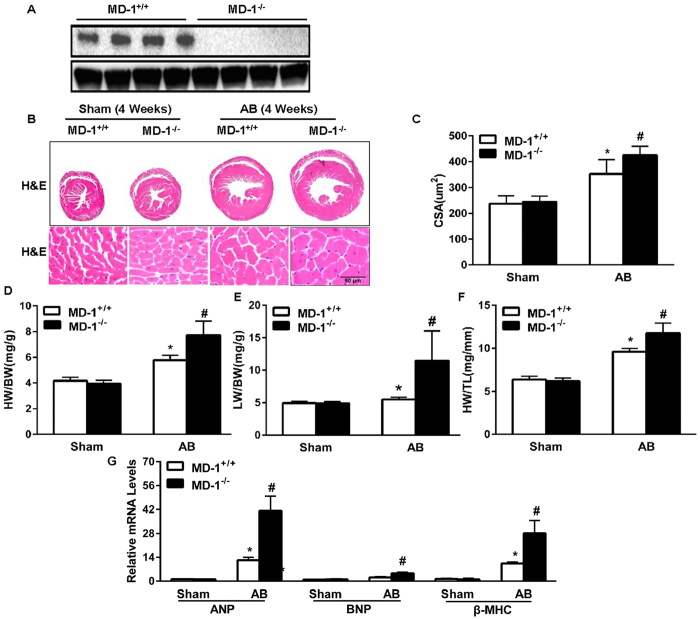
Effects of MD-1 deletion on cardiac hypertrophy. (**A**) MD-1 protein levels in samples obtained from MD-1^+/+^ and MD-1^−/−^ mice. (**B**) Gross hearts and HE staining were performed at 4 weeks after sham or AB surgery (n = 6). (**C**) Results of statistical analysis of the cell sectional areas (n = 100 + cells). (**D**–**F**) HW/BW, LW/BW, and HW/TL ratios for the indicated groups (n = 12). (**G**) Real-time PCR analyses of the hypertrophic markers ANP, BNP and β-MHC after AB in MD-1^+/+^ and MD-1^−/−^ mice (n = 4). *P < 0.05 vs. MD-1^+/+^/shams; ^#^P < 0.05 vs. MD-1^+/+^/AB.

**Figure 4:**
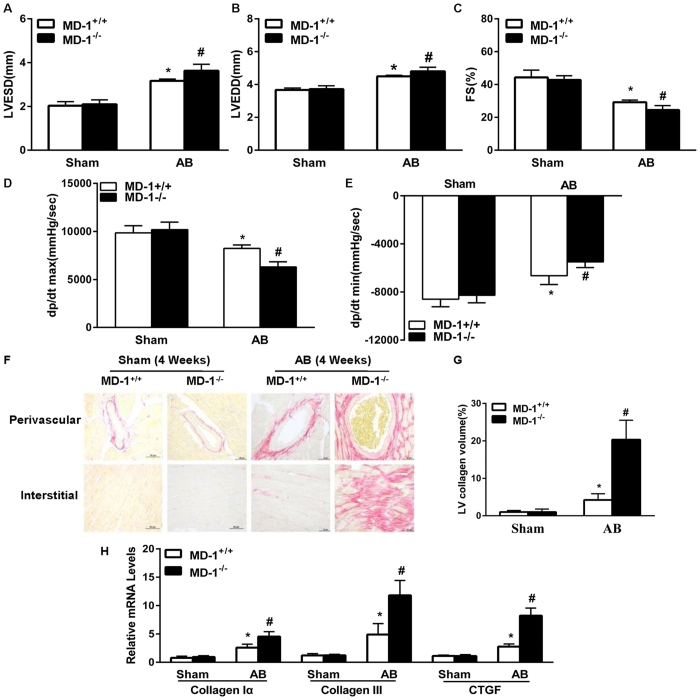
Effects of MD-1 disruption on cardiac hypertrophy. (**A**–**C**) Echocardiographic results for MD-1^+/+^ and KO mice (n = 12). (**D**,**E**) Haemodynamic parameters of MD-1^+/+^ and MD-1^−/−^ mice at 4 weeks after AB (n = 6–9). (**F**) PSR staining of histological sections of LVs was performed for the indicated groups at 4 weeks after AB (n = 6). (**G**) The fibrotic areas of the histological sections were quantified using an image analysis system (n = 28–30 fields). (**H**) Real-time PCR analyses of the mRNA levels of the fibrotic markers collagen Iα, collagen III and CTGF in the indicated mice (n = 4). *P < 0.05 vs. MD-1^+/+^/shams; ^#^P < 0.05 vs. MD-1^+/+^/AB.

To evaluate the role of endogenous MD-1 in cardiac hypertrophy, we performed AB on MD-1^−/−^ and MD-1^+/+^ mice. The absence of MD-1 in the murine hearts was confirmed by immunoblotting (Fig. 3A). After 4 weeks, the MD-1^+/+^ mouse hearts were significantly enlarged, and the MD-1^+/+^ cardiomyocyte sizes were increased (Fig. 3B,C). In addition, the heart weight to body weight (HW/BW), lung weight to body weight (LW/BW), and heart weight to tibia length (HW/TL) ratios were higher in the MD-1^+/+^ mice than in sham-operated mice (Fig. 3D–F). However, remarkably, at 4 weeks after AB, these parameters were more pronounced in MD-1^−/−^ mice than in AB-treated MD-1^+/+^ mice (Fig. 3B–F). Consistent with these results, the mRNA levels of several markers of hypertrophy, including ANP, BNP, and β-MHC, were significantly higher in the knockout hearts than in the MD-1^+/+^ hearts at 4 weeks after AB (Fig. 3G). Furthermore, the MD-1^−/−^ mice exhibited increased cardiac dilation and dysfunction, as demonstrated by echocardiographic and haemodynamic measurements, including left ventricular end diastolic diameter (LVEDD), left ventricular end systolic diameter (LVESD), fractional shortening (FS), and the maximum and minimum rates of pressure increase in isovolumetric LV contractions (dp/dt max and dp/dt min, respectively) (Fig. 4A–E). To further explore the effects of MD-1 deletion on maladaptive cardiac remodelling, we examined cardiac fibrosis. At 4 weeks after AB, we observed dramatic increases in perivascular and interstitial fibrosis in the MD-1^+/+^ hearts, and these increases were further aggravated in the MD-1^−/−^ hearts (Fig. 4F,G). Accordingly, at 4 weeks after AB, the MD-1^−/−^ hearts exhibited significant increases in the total collagen volume (Fig. 4F,G) and mRNA levels of procollagen type I a1 (collagen Iα), procollagen type III (collagen III), and connective tissue growth factor (CTGF), which are known mediators of fibrosis (Fig. 4H), compared with the MD-1^+/+^ hearts. Collectively, these results provide the first *in vivo* evidence indicating that the loss of MD-1 in the heart contributes to the maladaptive response to chronic pressure overload.

### Overexpression of MD-1 attenuates AB-induced cardiac remodelling

**Figure 5:**
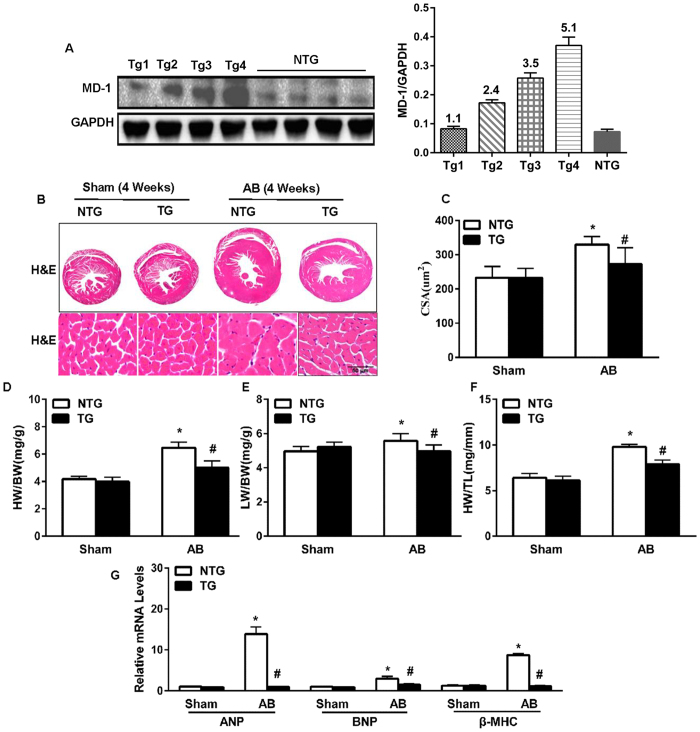
The effects of MD-1 on cardiac hypertrophy and cardiac performance *in vivo*. (**A**) Representative western blots and results of quantitative analysis of the transgenic MD-1 levels in heart tissues from four lines of TG mice and control WT mice. (**B**) Gross heart and HE staining were performed at 4 weeks after AB for NTG and TG mice (n = 6). (**C**) Results of statistical analysis of the myocyte cross-sectional areas (n = 100 + cells). (**D**–**F**) HW/BW, LW/BW, and HW/TL ratios for the indicated groups (n = 12). (**G**) Real-time PCR analyses of the mRNA levels of the hypertrophic markers ANP, BNP and β-MHC induced by AB in the indicated mice (n = 4 mice per experimental group). *P < 0.05 vs. NTG/shams; ^#^P < 0.05 vs. NTG/AB.

**Figure 6:**
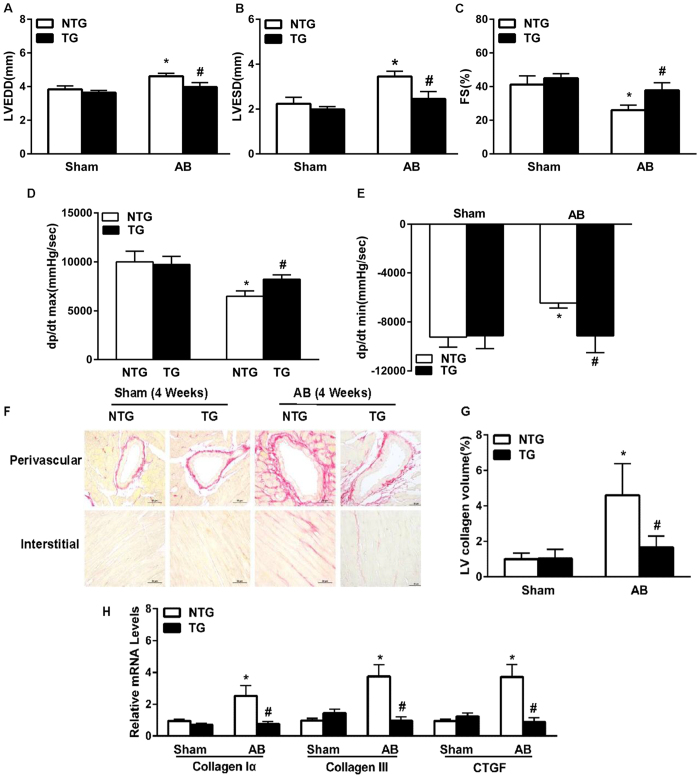
The effects of MD-1 on cardiac hypertrophy and cardiac performance *in vivo*. (**A**–**C**) Echocardiography analysis of myocardial contractile function in NTG and TG mice (n = 12). (**D**,**E**) Haemodynamic parameters of TG and NTG mice at 4 weeks after AB (n = 6–9). (**F**) PSR staining of histological sections of LVs was performed for the indicated groups at 4 weeks after AB (n = 5). (**G**) Fibrotic areas of the histological sections were quantified using an image analysis system (n = 26–28 fields). (**H**) Real-time PCR analyses of the mRNA levels of the fibrotic markers collagen Iα, collagen III, and CTGF in the indicated mice (n = 4 mice per experimental group). *P < 0.05 vs. NTG/shams; ^#^P < 0.05 vs. NTG/AB.

To further examine the function of endogenous MD-1 in the mouse heart *in vivo*, TG mice overexpressing cardiac-specific human MD-1 were generated using the α-myosin heavy chain (α-MHC) promoter. Western blotting analyses revealed that MD-1 was successfully overexpressed in the hearts of four generated TG mouse lines (Fig. 5A). At baseline, all MD-1-TG mice were healthy and exhibited normal cardiac morphology and contractile function. We then selected the highest-expressing MD-1 line (TG4) for further experiments. The MD-1-TG mice and their wild-type (WT) littermates (referred to as “NTG” hereafter) were subjected to AB surgery or sham operation. At 4 weeks post-operation, the TG mice exhibited significantly less hypertrophy than the NTG mice, as demonstrated by reductions in cardiomyocyte size (in gross hearts and after haematoxylin and eosin (HE) staining, Fig. 5B,C) and the HW/BW, HW/TL, and LW/BW ratios (Fig. 5D–F). No significant differences in these parameters were observed between the sham-operated TG and NTG mice. In addition, the AB-induced activation of hypertrophy markers (ANP, BNP, and β-MHC) was also markedly decreased in the TG hearts compared with the NTG hearts (Fig. 5G). Furthermore, AB-induced cardiac dilation and dysfunction (as assessed by performing echocardiography and haemodynamic measurements) was substantially ameliorated in the MD-1-TG mice compared with the NTG mice (Fig. 6A–E). Accordingly, the extent of cardiac fibrosis triggered by chronic pressure overload was markedly reduced in the TG mice (Fig. 6F,G). Subsequent analyses of the mRNA levels of known mediators of fibrosis, including collagen Iα, collagen III and CTGF, revealed that the fibrotic response was lower in the TG mice than in the control NTG mice (Fig. 6H). Taken together, these gain-of-function data indicate that the up-regulation of MD-1 protects hearts against pressure overload-induced remodelling.

### MD-1 negatively regulates MEK-ERK1/2 and the NF-κB signalling pathway in hypertrophic hearts and cardiomyocytes

**Figure 7:**
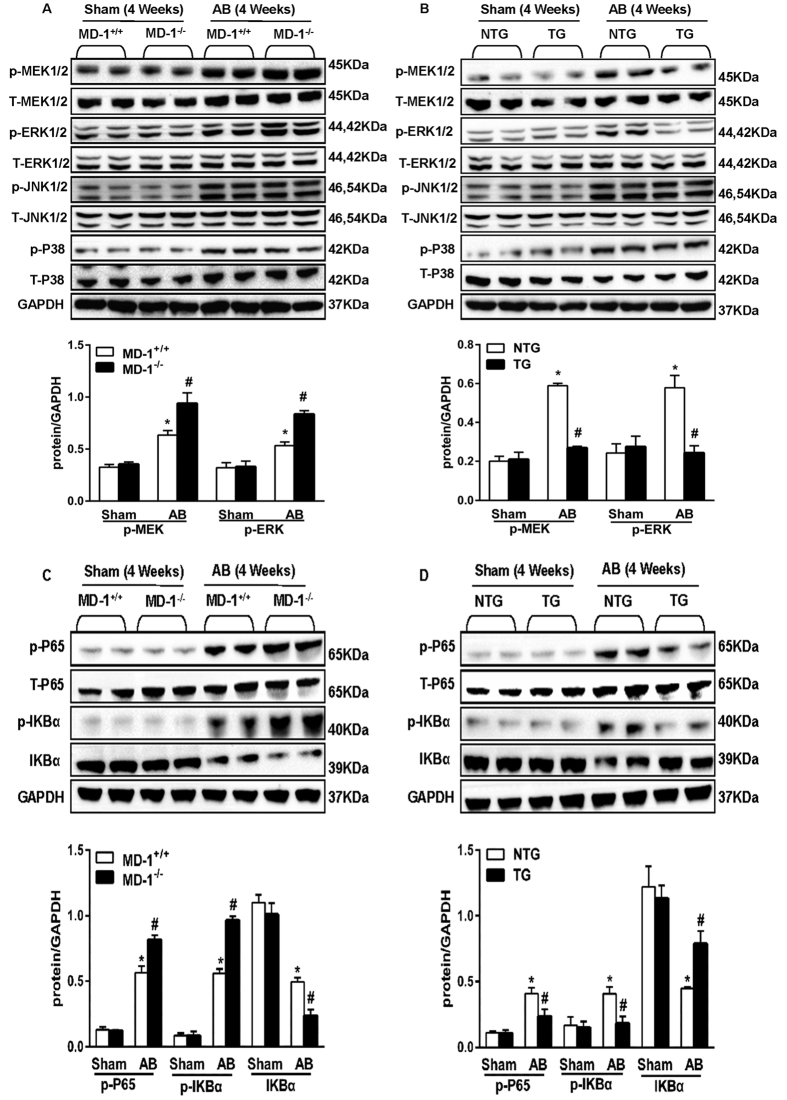
Effects of MD-1 on MEK-ERK1/2 and NF-κB signalling pathways *in vivo*. (**A**,**C**) Representative western blots and results of quantitative analysis of the phosphorylated and total protein levels of MEK1/2, ERK1/2, JNK1/2, P38, P65 and IkBα in MD-1^−/−^ and MD-1^+/+^ mice at 4 weeks after sham or AB surgery (n = 4). *P < 0.05 vs. MD-1^+/+^/shams; ^#^P < 0.05 VS. MD-1^+/+^/AB. (**B**,**D**) Representative western blots and results of quantitative analysis of the phosphorylated and total protein levels of MEK1/2, ERK1/2, JNK1/2, P38, P65 and IkBα in TG and NTG mice at 4 weeks after sham or AB surgery (n = 4). *P < 0.05 vs. NTG/shams; ^#^P < 0.05 vs. NTG/AB.

**Figure 8:**
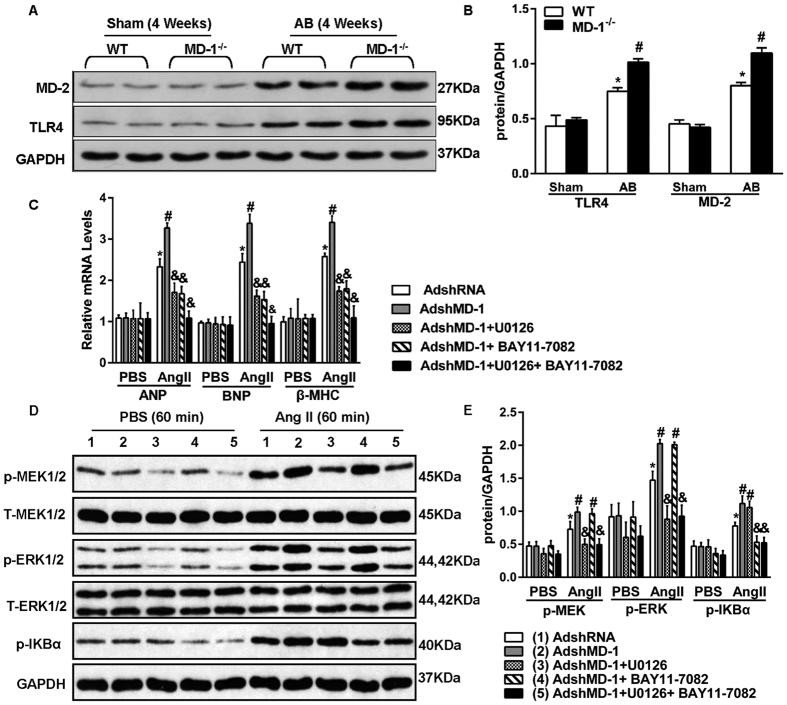
Inactivation of MEK–ERK1/2 and NF-κB signalling rescues the adverse effects of MD-1 deficiency on Ang II-induced myocyte hypertrophy. (**A**,**B**) Representative western blot and results of quantitative analysis of the TLR4 and MD-2 protein levels in WT and MD-1^−/−^ mice at 4 weeks after sham or AB surgery (n = 4). *P < 0.05 vs. WT/shams; #P < 0.05 vs. WT/AB. (**C**) Real-time RT-PCR analysis of the mRNA levels of ANP, BNP and β-MHC in neonatal rat cardiomyocytes infected with AdshMD-1 and U0126 or BAY11–7082 in response to Ang II. PBS was used as a control solution. Similar results were obtained from three independent experiments. (**D**,**E**) Representative western blots and results of quantitative analysis of the phosphorylated MEK1/2, ERK1/2, and IkBα protein levels following infection with AdshMD-1 and treatment with Ang II in the presence or absence of U0126 or BAY11-7082 (n = 4). *P < 0.05 vs. PBS controls; ^#^P < 0.05 vs. AdshRNA/Ang II; ^&^P < 0.05 vs. AdshMD-1/Ang II.

Because MD-1 is an inhibitor of the TLR4 signalling pathway, we investigated the possible involvement of the MAPK and NF-κB pathways in MD-1-mediated cardioprotective effects. Western blot analyses demonstrated that AB significantly increased the levels of phosphorylated MEK1/2, ERK1/2, JNK1/2, p38, p65 and IKBα in both MD-1^−/−^ and MD-1^+/+^ hearts after 4 weeks of AB (Fig. 7A,C). However, MEK1/2 and ERK1/2 activation was significantly enhanced in the MD-1^−/−^ hearts compared with the MD-1^+/+^ hearts, although similar levels of JNK1/2 and p38 activation were observed in these two groups (Fig. 7A). Similarly, gain-of-function experiments revealed that the AB-induced activation of MEK1/2, ERK1/2, p65 and IKBα was almost completely blocked in MD-1-TG hearts (Fig. 7B,D). To confirm these findings and exclude potential *in vivo* compensatory mechanisms, we further examined the effects of MD-1 on MEK-ERK1/2 and NF-κB signalling using cultured NRCMs. NRCMs were infected with AdshMD-1 to knockdown MD-1 or with AdMD-1 to overexpress MD-1, and they were then treated with 1 μM Ang II for 60 min. In accordance with the *in vivo* findings, Ang II-induced activation of MEK1/2, ERK1/2, p65 and IKBα was enhanced in the MD-1-knockdown cells but attenuated in the MD-1-overexpressing cells (see Supplementary Fig. S1A–D). To further explore the relationship between MD-1 and the TLR4 signalling pathway, we measured the levels of TLR4 and MD-2 in both WT and MD-1^−/−^ hearts after 4 weeks of AB. Western blot analyses showed that AB significantly increased the levels of TLR4 and MD-2 in both the WT and MD-1^−/−^ hearts after 4 weeks of AB (Fig. 8A,B). However, the expression levels of TLR4 and MD-2 were much higher in MD-1^−/−^ hearts than in WT hearts (Fig. 8A,B). Taken together, these findings suggest that MD-1-elicited cardioprotective effects are associated with inhibition of the MEK–ERK1/2 and NF-κB signalling cascades in hearts subjected to pressure overload.

### Inactivation of MEK–ERK1/2 and NF-κB signalling rescues the adverse effect of MD-1 deficiency on Ang II-induced myocyte hypertrophy

The aforementioned results suggest that the inactivation of ERK1/2 and NF-κB rescues effects of accelerating hypertrophy induced by MD-1 knockdown. To test this hypothesis, we exposed cultured NRCMs that had been previously infected with AdshMD-1 to an ERK1/2 inhibitor, U0126, or an NF-κB inhibitor, BAY11–7082, and then added Ang II for 48 h. Analysis of the mRNA levels of the hypertrophy markers (ANP, BNP and β-MHC) revealed that U0126 treatment significantly reversed the deteriorative effects of MD-1 deficiency on the hypertrophy of Ang II-treated cells compared with that of PBS-treated controls (Fig. 8C). Similar results were observed for the BAY11-7082 treatment (Fig. 8C). Interestingly, these effects were more pronounced in the cells pretreated with U0126 and BAY11-7082 (Fig. 8C). Next, to investigate whether cross-talk occurs between activation of MEK-ERK1/2 and the NF-κB signalling pathway, we examined the protein levels of p-MEK1/2, p-ERK1/2 and p-IkBα. As shown in Fig. 8D,E, treatment of Ang II-induced NRCMs with the MEK-ERK1/2 inhibitor U0126 resulted in significant inhibition of the expression and activity of MEK-ERK1/2. However, it had no effect on the expression of p-IkBα. Similar results were observed in NRCMs treated with the NF-κB inhibitor BAY11-7082. These data suggest that MD-1-mediated cardioprotective effects are largely dependent on the inhibition of MEK–ERK1/2 and NF-κB signalling.

## Discussion

In the present study, we used both gain-of-function and loss-of function approaches to decipher the potential role of MD-1 in pathological cardiac hypertrophy. For the first time, we observed that overexpression of MD-1 in mouse hearts attenuated chronic pressure overload-induced cardiac remodelling, whereas loss of MD-1 promoted the development of pathological cardiac hypertrophy. We also found that MD-1 expression was significantly reduced in the hypertrophic hearts. Thus, MD-1 up-regulation may provide a new therapeutic strategy for the treatment of pathological cardiac hypertrophy.

MD-1, also known as Ly86, a secreted protein that interacts with RP105^15^, plays an important role in the TLR4 signalling pathway^16^. Previous studies have demonstrated the potential involvement of MD-1 in the (patho) physiological regulation of the innate immune system and inflammation^25^,^26^,^27^. For example, Gorczynski *et al*.^25^ have implicated the gene expression of murine MD-1 in the regulation of subsequent T cell activation and cytokine production. In addition, Huaqin *et al*.^28^ have demonstrated that MD-1 deficiency attenuates dextran sodium sulphate (DSS)-induced colitis. Recent evidence suggests that the RP105/MD-1 complex contributes to high-fat diet (HFD)-induced obesity, adipose tissue inflammation, and insulin resistance^29^. Therefore, we further examined the levels of inflammation cytokines in AB-treated MD-1^−/−^ and WT hearts. The results revealed that the mRNA levels of interleukin-6 (IL-6), interleukin-1β(IL-1β), tumour necrosis factor-α(TNF-α) and monocyte chemotactic protein 1 (MCP-1) were significantly increased in the hearts from both groups (WT and MD-1^−/−^) after 1 week of AB and that they were decreased to the basal levels after 4 weeks of AB; however, no significant differences were observed between the two groups. We also examined the mRNA levels of TLR4, MD2, RP105, and MD-1. The results showed that the mRNA levels of TLR4 and MD-2 were progressively increased in the hearts from both groups after AB but that the mRNA levels of RP105 and MD-1 were gradually decreased after AB (see Supplementary Fig. S2A–H). These data suggest that inflammatory response is prominent only during early phase of AB, and MD-1 may not regulate inflammation in AB-treated murine hearts.

The possible mechanisms by which MD-1 regulates cardiac hypertrophy may be associated with its downstream targets in the TLR4 signalling pathway. Thus, we examined the expression of MAPKs and NF-κB signalling. It is well known that the MAPK signalling cascade includes three major protein kinases, namely ERKs, JNKs, and p38, and that it is initiated in cardiac myocytes by stress stimuli. Activation of the MAPK cascade leads to the phosphorylation of numerous intracellular transcription factors, resulting in the reprogramming of cardiac gene expression. The precise role of MEK1-ERK1/2 in the regulation of cardiac hypertrophy remains under debate^7^,^30^. Studies have shown that ERK activation prevents cardiac hypertrophy^30^,^31^,^32^. However, other studies have demonstrated that hypertrophic stimuli activate ERK1/2 and induce hypertrophic responses^33^,^34^. In addition, constitutive activation of ERK1/2 signalling in the heart through expression of activated MEK1 has been reported to induce LV hypertrophy^35^. Further, a study involving the treatment of cells with the MEK1 inhibitor U0126 has demonstrated that ERKs are required for the hypertrophic response induced by Ang II and ET-1^34^,^36^,^37^. In the present study, we found that activation of both MEK1/2 and ERK1/2 was enhanced by the loss of MD-1 but that it was blocked by cardiac MD-1 overexpression in response to chronic pressure overload. However, MD-1 did not affect the phosphorylation of p38 or JNK1/2. More importantly, rescue experiments demonstrated that the inhibition of MEK-ERK1/2 signalling suppressed the accelerating effects of MD-1 knockdown on Ang II-induced cell hypertrophy. Taken together, these findings support the notion that MD-1 negatively regulates pathological cardiac hypertrophy partly by inhibiting the MEK-ERK1/2 signalling axis.

NF-κB is a critical transcription factor that functions downstream of the TLR4 signalling pathway^38^. Recent studies have suggested that the NF-κB signalling pathway plays a crucial role in the progression of cardiac hypertrophy^8^,^39^,^40^. Nonetheless, there are also some controversial reports on the role of NF-κB signalling in the regulation of cardiac hypertrophy. Some investigators have shown that NF-κB activation is required for the development of cardiac hypertrophy and cardiac dysfunction^41^,^42^. For example, cardiac-specific NF-κB inhibition mediated by expression of a stabilized IκBα mutant has been demonstrated to attenuate Ang II-induced cardiac hypertrophy^42^. In contrast, several other studies have disputed the importance of NF-κB in the regulation of cardiac hypertrophy^43^,^44^. For example, Hikoso *et al*.^43^ have reported that the cardiac-specific knockdown of IKKβ promotes cardiac hypertrophy, dilation, and dysfunction in response to pressure overload. In the present study, we observed that NF-κB activation was inhibited in hearts with MD-1 overexpression, suggesting that MD-1 may attenuate cardiac remodelling through the negative regulation of NF-κB signalling. More importantly, inhibition of NF-κB signalling by the NF-κB inhibitor BAY11-7082 ameliorated the deteriorative effects of MD-1 knockout on Ang II-induced cell hypertrophy. Therefore, the inhibitory effects of MD-1 on cardiac hypertrophy may be largely dependent on the inactivation of NF-κB signalling.

Next, we examined the cross-talk that occurs between the MEK-ERK and NF-κB signalling pathways. Currently, some controversy exists regarding the cross-talk between these pathways. One study has shown that MEK activation causes phosphorylation of NF-κB and activation of the NF-κB pathway in osteoclasts^45^. However, another study has reported that the MEK-1/ERK inhibitor PD98059 does not affect antiphospholipid antibody-induced NF-κB binding activity^46^. In the present study, we observed no cross-talk between the MEK-1/ERK and NF-κB signalling pathways. These results suggest that the MD-1-mediated activation of both the NF-κB pathway and MAPK signalling contribute to the development of cardiac hypertrophy. Moreover, NF-κB pathway activation is independent of the MEK-ERK signalling pathway.

In conclusion, our data have demonstrated that MD-1 plays a crucial role in protecting against cardiac hypertrophy and fibrosis in response to hypertrophic stimuli by inhibiting the MEK-ERK1/2 and NF-κB signalling pathways. Our study provides insights into the mechanisms underlying cardiac hypertrophy and may have significant implications for the development of novel strategies to treat cardiac hypertrophy by targeting MD-1.

## Materials and Methods

### Drugs and reagents

The following antibodies were purchased for use in the western blot experiments: P-JNK1/2 (Cell Signalling; 4668S); t-JNK1/2 (Cell Signalling; 9258P); p-MEK1/2 (Cell Signalling; 9154); t-MEK1/2 (Cell Signalling; 9122); p-ERK1/2 (Cell Signalling; 4370P); t-ERK1/2 (Cell Signalling; 4695P); p-p38 (Cell Signalling; 4511P); t-p38 (Cell Signalling; 8690P); p-IκBαSer32/36 (Cell Signalling; 9246); IκBα (Cell Signalling; 4814); p-p65Ser536 (Cell Signalling; 3033); p65 (Cell Signalling; 4764); GAPDH (Cell Signalling; 2118S); ANP (Santa Cruz Biotechnology; SC-20158); MYH7 (Santa Cruz Biotechnology; SC-53089); MD-1 (Santa Cruz Biotechnology; SC-390613); RP105 (Santa Cruz Biotechnology; SC-27841); TLR4 (Santa Cruz Biotechnology; SC-293072); MD-2 (Novus Biologicals; NB100-56655); Ang II (Sigma; F3165); and U0126 and BAY11-7082 (Selleckchem). Foetal calf serum (FCS) was obtained from Gibco. Cell culture reagents and all other reagents were obtained from Sigma.

### Human heart samples

All procedures involving human tissue samples were approved by the Human Research Ethics Committee of Renmin Hospital of Wuhan University, Wuhan, China. This study conformed to the principles outlined in the Declaration of Helsinki. Informed consent was obtained from the families of prospective heart donors. Samples were collected from the LVs of failing human hearts of patients with HCM undergoing heart transplantation. Control samples were obtained from the LVs of healthy heart donors who died in accidents but whose hearts were not suitable for transplantation for non-cardiac reasons.

### Study animals

All experiments involving animals were approved by the Animal Care and Use Committee of Renmin Hospital of Wuhan University and conformed to the Guide for the Care and Use of Laboratory Animals published by the US National Institutes of Health (NIH Publication No. 85–23, revised 1996). All of the animals were housed in an environment with a controlled light cycle (12 h light/12 h dark) and constant temperature and humidity, and food and water were provided *ad libitum*. All of the animal experiments were performed by an experimenter who was blinded to the animals’ genotypes.

### Animals and animal models

All protocols were approved by the Animal Care and Use Committee of Renmin Hospital of Wuhan University. A human MD-1 cDNA construct containing the full-length human MD-1 cDNA was cloned downstream of the human cardiac α-MHC promoter. TG mice were produced by microinjection of the α-MHC-MD-1 construct into fertilized mouse embryos (C57BL/6 J background). TG mice were identified by polymerase chain reaction (PCR) and western blot analyses. Male MD-1 knockout mice (MD-1^−/−^ mice) were purchased from the Japan RIKEN BioResource Centre Mouse (BRC) (B6.129P2-MD-1 <tm1Kmiy>). The deletion of MD-1 was confirmed in the obtained MD-1-KO mice by western blot analyses of heart tissues. AB was performed as previously described^47^. In addition, Doppler analysis was conducted to ensure that adequate constriction of the aorta had been induced. The wall thickness and internal diameter of the LV were assessed by echocardiography at the indicated time points after surgery.

### Blood pressure and echocardiography

A microtip catheter transducer (SPR-839; Millar Instruments) was inserted into the right carotid artery and advanced into the LV. After allowing the animal to stabilize for 15 min, an ARIA pressure-volume conductance system coupled to a Powerlab/4SP A/D converter that stored and displayed data on a personal computer was used to continuously record pressure signals and heart rate, as previously described^48^. Echocardiography was performed using a Mylab30CV (Esaote S.p.A.) instrument with a 10 MHz linear array ultrasound transducer. LV was assessed along both the parasternal long axis and short axis views at a frame rate of 120 Hz. End-systolic and end-diastolic measurements were defined and assessed as the phases with the smallest and largest LV areas, respectively. LVEDD and LVESD were measured using LV M-mode tracing with a sweep speed of 50 mm/s at the level of the midpapillary muscle. Subsequently, the percentage of left ventricular fractional shortening (LVFS) was calculated as (LVEDD-LVESD)/LVEDD × 100%.

At the end of the experiment, the hearts and lungs of the sacrificed mice were dissected and weighed to compare the HW/BW (in mg/g), LW/BW (in mg/g), and HW/TL (in mg/cm) ratios.

### Histological analysis

Hearts were excised, washed with saline solution and placed in 10% formalin. They were then transversely cut close to the apex to visualize the left and right ventricles. Several sections (4–5 mm thick) were prepared. HE-stained sections were used to determine the cross-sectional areas of myocytes. Single myocytes were measured using a quantitative digital image analysis system (Image Pro-Plus, version 6.0). The outlines of no fewer than 100 myocytes were traced for each group. Evidence of interstitial and perivascular collagen deposition was visualized using Picro Sirius Red (PSR), and high-magnification light micrographs were then obtained by light microscopy.

### Adenoviral vector infection and NRCM culturing

To overexpress MD-1, we used a replication-defective adenoviral vector containing the entire coding region of the MD-1 gene under control of the cytomegalovirus promoter. A similar adenoviral vector encoding the GFP gene was used as a control. Using three rat short hairpin (sh) MD-1 constructs (purchased from HanBio, Shanghai, China), we then generated three AdshMD-1 adenoviruses and selected the one that caused the most significant decrease in the MD-1 level for use in further experiments. AdshRNA was used as a control. We infected cardiomyocytes with AdMD-1 or AdGFP, as well as AdshMD-1 or AdshRNA, at a multiplicity of infection of 100. The results showed that 95% to 100% of the cells expressed the transgenes with no detectable toxic effects.

Primary NRCMs were cultured as previously described^19^. Briefly, cells from the hearts of 1- to 2-day-old Sprague-Dawley rats were seeded at a density of 1 × 10^5^ cells/well on gelatine-coated, six-well culture dishes. The cells were then cultured in Dulbecco’s Modified Eagles Medium (DMEM)/F12 medium containing 10% FCS, BrdU (0.1 mM), and penicillin/streptomycin at 37 °C for 48 h. The culture medium was subsequently changed to serum-free DMEM/F12, and the cells were incubated for 12 h. Then, the cells were treated with adenovirus and/or Ang II (1 μM), U0126 (10 μM) or BAY11-7082 (10 μM) for 30 min. Protein synthesis was evaluated in the cultured cells by western blot analysis. To obtain CSA measurements, the cardiomyocytes were stained with α-actin and DAPI. Microscopic images were captured, and the surface areas of the myocytes were measured using a quantitative digital image analysis system.

### Real-time PCR

Tissues were frozen in liquid nitrogen for RNA and protein analyses. For quantitative real-time PCR (qRT-PCR), total RNA was extracted from the LVs using TRIzol reagent. Real-time PCR (RT-PCR) analyses were conducted to determine the mRNA expression levels of ANP, BNP, β-MHC, collagen Iα, collagen III, CTGF, IL-6, IL-1β, TNF-α, MCP-1, TLR4, MD2, RP105, and MD-1 in the hearts from the different groups. The calculated mRNA levels were normalized by the GAPDH mRNA level.

### Protein extraction and western blot analysis

The protein concentrations of the samples were determined using the Bradford method. Lysates were resolved using sodium dodecyl sulphate-polyacrylamide gel electrophoresis (SDS-PAGE), and then the proteins were transferred to nitrocellulose membranes, which were subsequently blocked in 5% non-fat dry milk in Tris-buffered saline/Tween. The membranes were then incubated with the primary antibodies overnight at 4 °C, followed by incubation with the peroxidase-conjugated secondary antibodies for 2 h. The separated proteins were visualized using an enhanced chemiluminescence system. GAPDH was used as a loading control.

### Statistical analysis

All data are expressed as the mean ± SEM. Differences among groups were assessed by two-way ANOVA, followed by post hoc Tukey’s test. Comparisons between two groups were analysed using unpaired Student’s t-test. A P < 0.05 was considered to indicate a significant difference.

## Additional Information

**How to cite this article**: Xiong, X. *et al*. Novel Protective Role of Myeloid Differentiation 1 in Pathological Cardiac Remodelling. *Sci. Rep.* **7**, 41857; doi: 10.1038/srep41857 (2017).

**Publisher's note:** Springer Nature remains neutral with regard to jurisdictional claims in published maps and institutional affiliations.

## Supplementary Material

Supplementary Information
