# Supplementary material for: Novel Protective Role of Myeloid Differentiation 1 in Pathological Cardiac Remodelling
Source: Sci Rep. 2017 Feb 6;7:41857. doi: 10.1038/srep41857 (PMC5292962; doi:10.1038/srep41857)
Supplement: Supplementary Information [file srep41857-s1.pdf]

Novel Protective Role of Myeloid Differentiation 1 in Pathological Cardiac Remodelling

Xiaojv Xiong [1, 2, 3], Yu Liu [1, 2, 3], Yang Mei [1, 2,3], Jianye Peng [1,2,3], Zhiqiang Wang[1, 2, 3,] Bin Kong [1, 2, 3], Peng Zhong [1, 2, 3], Liang Xiong [1, 2, 3], Dajun Quan [1, 2, 3], Qi Li [1, 2, 3], Guangji Wang [1, 2, 3], He Huang [1, 2, 3*]

[1] Department of Cardiology, Renmin Hospital of Wuhan University, Wuhan, Hubei Province, PR China

[2] Cardiovascular Research Institute of Wuhan University, Wuhan, Hubei Province, PR China

[3] Hubei Key Laboratory of Cardiology, Wuhan 430060, Hubei Province, PR China;

***Xiaojv Xiong and Yu Liu are co-first authors**

Correspondence to

He Huang, MD

Department of Cardiology, Renmin Hospital of Wuhan University

Cardiovascular Research Institute, Wuhan University,

Hubei Key Laboratory of Cardiology,

Jiefang Road 238, Wuhan 430060, PR China.

Tel/fax: +86 27 88042922.

E-mail: huanghe1978@hotmail.com

Supplemental Figures

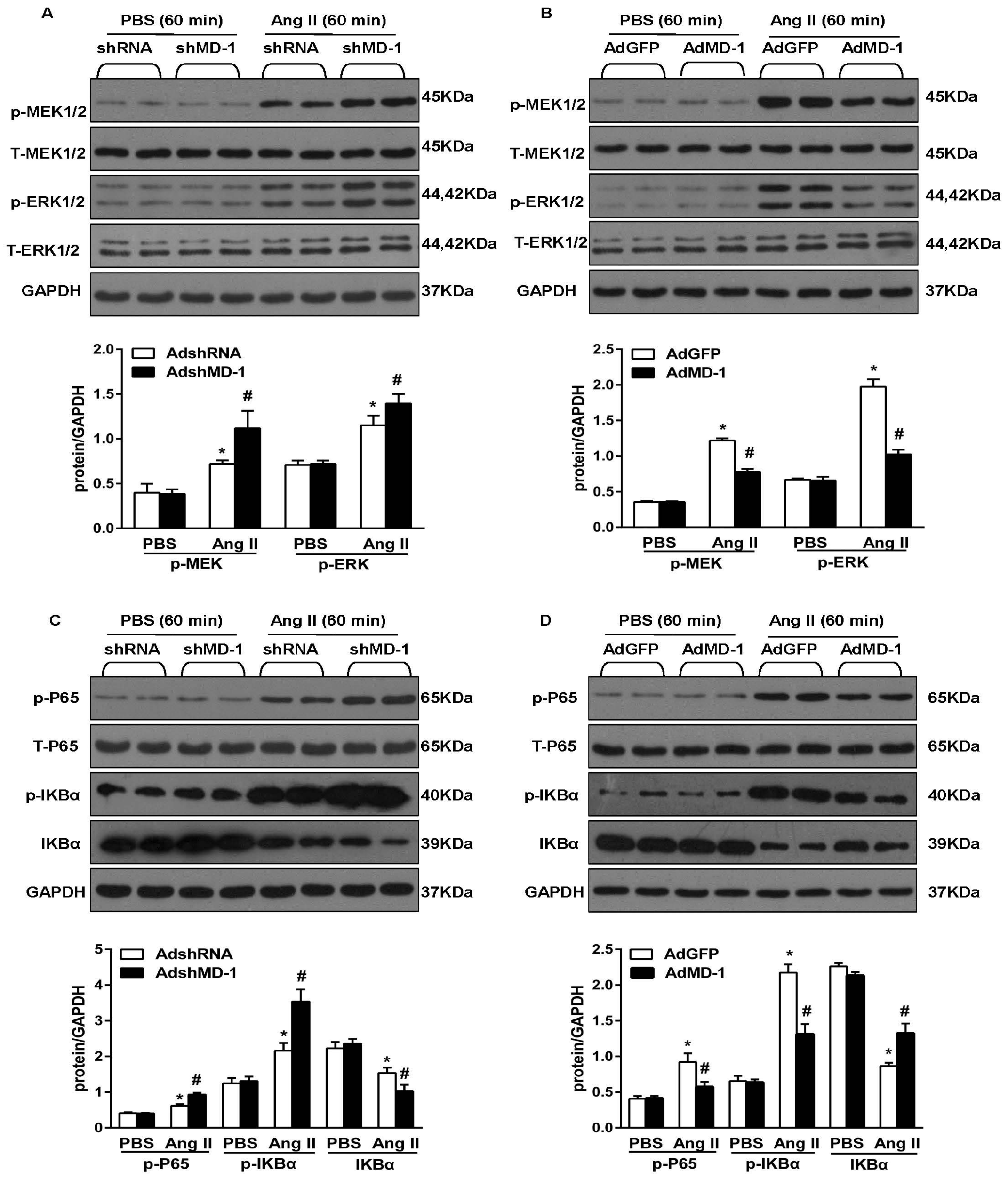

Figure S1 Effects of MD-1 on MEK-ERK1/2 and NF-κB signalling pathways in vitro. (A, C) Representative blots and results of quantitative analysis of the phosphorylated and total protein levels of MEK1/2, ERK1/2, P65 and IkBα following infection with AdshMD-1 and treatment with PBS or Ang II (n=4). *P＜0 .05 vs. AdshRNA/PBS; #P＜0 .05 vs. AdshRNA/Ang II. **(B, D)** Representative blots and results of quantitative analysis of the phosphorylated and total protein levels of MEK1/2, ERK1/2, P65 and IkBα following infection with AdMD-1 in response to PBS or Ang II (n=4). *P＜0 .05 vs. AdGFP/PBS; #P＜0 .05 vs. AdGFP/Ang II. GAPDH was used as a loading control.

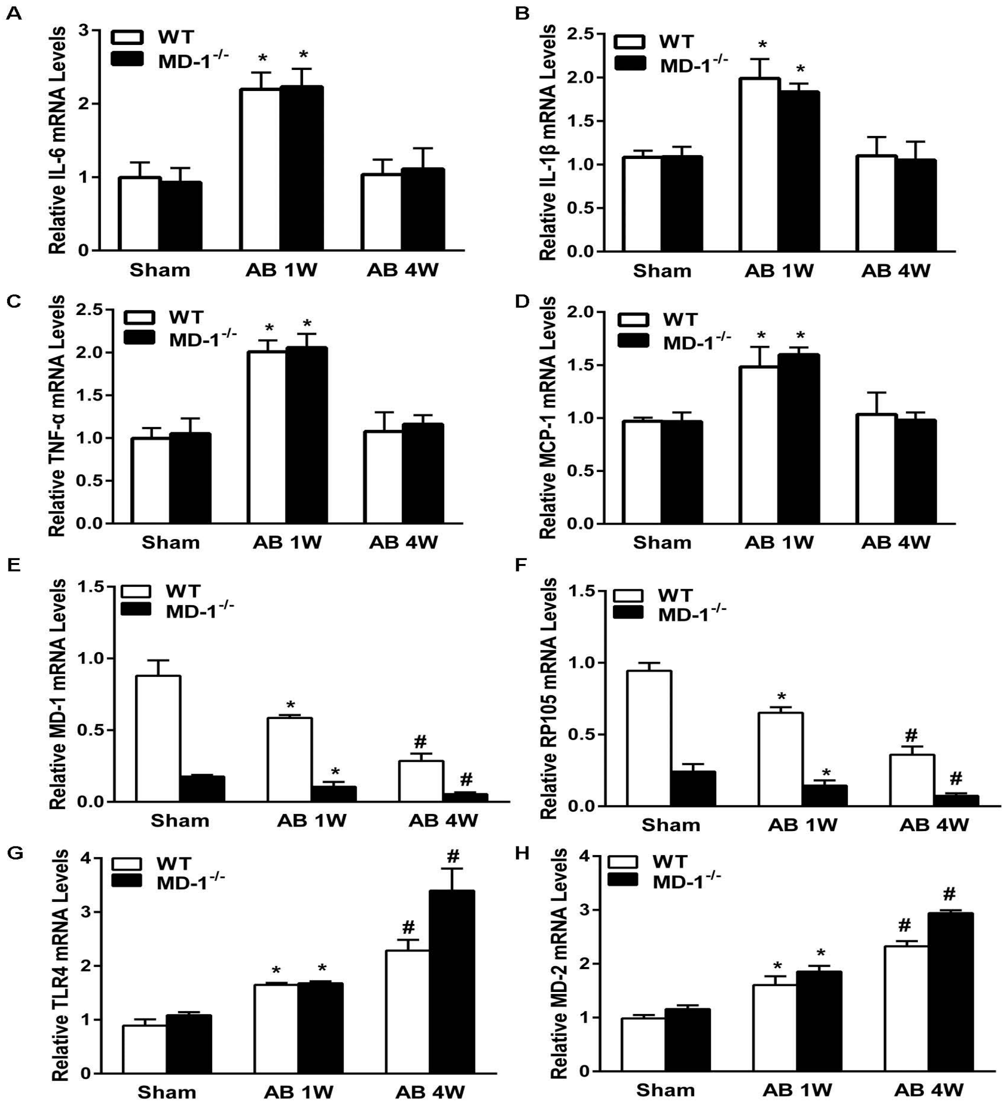

Figure S2 Effects of MD-1 on mRNA levels of inflammatory cytokines, TLR4-MD2 and RP105-MD1 in WT vs. MD-1 KO hearts at week1 and at week4 after AB. **(A-D)** Relative **(A)** IL-6, **(B)** IL-1β, **(C)** TNF-α, and **(D)** MCP -1 mRNA levels in samples obtained from WT and MD-1$^{-/-}$ mice (n=4). **(E-H)** Relative **(E)** MD-1, **(F)** RP105, **(G)** TLR4, and **(H)** MD-2 mRNA levels in samples obtained from WT and MD-1$^{-/-}$ mice (n=4). *P $<$0 .05 vs. WT/shams; #P $<$0 .05 vs. AB 1W.
